# Organ-Specific Gene Expression Changes in the Fetal Liver and Placenta in Response to Maternal Folate Depletion

**DOI:** 10.3390/nu8100661

**Published:** 2016-10-22

**Authors:** Jill A. McKay, Long Xie, Michiel Adriaens, Chris T. Evelo, Dianne Ford, John C. Mathers

**Affiliations:** 1 Human Nutrition Research Centre, Institute for Health & Society, Newcastle University, Sir James Spence Institute, Royal Victoria Infirmary, Queen Victoria Road, Newcastle upon Tyne NE1 4LP, UK; jill.mckay@ncl.ac.uk; 2 Human Nutrition Research Centre; Institute of Cellular Medicine, Newcastle University, Newcastle upon Tyne NE4 5PL, UK; long.xie@ncl.ac.uk; 3 Maastricht Centre for Systems Biology, MaCSBio, Maastricht University, Maastricht 6200 MD, The Netherlands; michiel.adriaens@maastrichtuniversity.nl; 4 Department of Bioinformatics, BiGCaT, Maastricht University, Maastricht 6200 MD, The Netherlands; chris.evelo@maastrichtuniversity.nl; 5 Faculty of Health and Life Sciences, Northumbria University, Newcastle upon Tyne NE1 8ST, UK; dianne.ford@northumbria.ac.uk

**Keywords:** transcriptome, programming, developmental origins of health and disease, pregnancy, diet

## Abstract

Growing evidence supports the hypothesis that the in utero environment can have profound implications for fetal development and later life offspring health. Current theory suggests conditions experienced in utero prepare, or “programme”, the fetus for its anticipated post-natal environment. The mechanisms responsible for these programming events are poorly understood but are likely to involve gene expression changes. Folate is essential for normal fetal development and inadequate maternal folate supply during pregnancy has long term adverse effects for offspring. We tested the hypothesis that folate depletion during pregnancy alters offspring programming through altered gene expression. Female C57BL/6J mice were fed diets containing 2 mg or 0.4 mg folic acid/kg for 4 weeks before mating and throughout pregnancy. At 17.5 day gestation, genome-wide gene expression was measured in male fetal livers and placentas. In the fetal liver, 989 genes were expressed differentially (555 up-regulated, 434 down-regulated) in response to maternal folate depletion, with 460 genes expressed differentially (250 up-regulated, 255 down-regulated) in the placenta. Only 25 differentially expressed genes were common between organs. Maternal folate intake during pregnancy influences fetal gene expression in a highly organ specific manner which may reflect organ-specific functions.

## 1. Introduction

The developmental origins of health and disease (DOHaD) hypothesis proposes that in utero and early life exposures can lead to altered programming of offspring. Such programming events can cause permanent changes in organ development, physiology and metabolism leading to altered disease risk in later life. Suboptimal nutrition, both under- and over-nutrition, during fetal and neonatal development increases susceptibility to a wide range of diseases [1]. These observations indicate a degree of plasticity during development, in which the fetal phenotype may be altered due to environment cues [2] to prepare it for the anticipated post-natal environment [3]. The biological mechanisms underlying this “programming” effect of nutrition during early life are poorly understood, but are likely to involve changes in gene expression.

The protective effect of adequate dietary folate intake and of folic acid supplementation during pregnancy on risk of neural tube defects (NTDs) is well established [4,5]. Further, epidemiological evidence suggests that adequate dietary folate intake or supplementation with folic acid during pregnancy may reduce the risk of other congenital defects [6] and adverse pregnancy outcomes [7], as well enhancing neurodevelopment [8] and reducing the risk of severe language delay [9], autism [10,11] and some cancers (leukaemia [12,13,14], brain tumours [15,16] and neuroblastoma [17]) [18] in children. In rodent models, folate deficiency during pregnancy can cause spontaneous abortion, teratogenic effects in offspring, reduced litter number, and altered offspring body weight [19,20]. Sufficient maternal folate intake during pregnancy is essential for successful pregnancy outcomes/normal fetal development and for the long-term health of the offspring.

Folate, a B vitamin, is central to one-carbon metabolism which, in addition to formation of the universal methyl donor, *S*-adenosyl-methionine (SAM), interacts with several other cellular pathways including amino acid metabolism and biosynthesis of purine and pyrimidines [21]. SAM is critical for the methylation of biological molecules including DNA, lipids and proteins. Epigenetic mechanisms (including methylation of DNA and of histones) which regulate gene expression are susceptible to modification via altered SAM availability in response to changes in folate intake (reviewed in [22]). Therefore, we hypothesised that inadequate folate supply during pregnancy alters programming of the offspring via changes in gene expression and that this is responsible for the observed adverse effects of this maternal nutritional insult on pregnancy outcomes and offspring health in later life. Mammals respond to inadequate nutrient supply by prioritising allocation of nutrients to specific purposes [23,24] which may result in cell, tissue and organ differences in gene expression. Little is known about such inter-organ differences in fetal gene expression in response to restricted folate supply. We hypothesised that the fetal liver and placenta, which represent organs with long and short-term consequences for the developing animal, would be subject to distinctly different responses to folate depletion. To test these hypotheses, we quantified genome-wide gene expression patterns in fetal liver and placenta in response to maternal folate depletion before, and throughout, pregnancy.

## 2. Experimental Section

### 2.1. Animal Husbandry and Experimental Diets

All animal procedures were approved by the Newcastle University Ethics Review Committee and the UK Home Office (Project Licence number 60/3979) and have been described previously [25]. Animals were housed in the Comparative Biology Centre, Newcastle University at 20–22 °C and with 12 h light and dark cycles. Fresh water was available ad libitum. Female C57BL/6J mice were allocated at random to either a low folate (0.4 mg folic acid/kg diet) or normal folate diet (2 mg folic acid/kg diet) (6 g of allocated diet was offered to each mouse per day), and maintained on this diet for 4 weeks prior to mating. Diet compositions were modified from AIN-93G [26] and have been described previously [25]. l-amino acids were used as a protein substitute. All ingredients, other than folic acid, were included in both diets at the same concentrations to avoid potential confounding through other dietary factors. The degree of folate depletion induced by feeding the diet containing 0.4 mg folic acid/kg was sufficient to impose a nutritional stress (evidenced by reduced circulating concentrations of folate) but not so severe as to limit reproduction. The normal folate diet contained 2 mg folic acid/kg diet which is considered sufficient to support breeding and maintenance in this species. Mice were time mated i.e., a male was added to a cage containing two females overnight and removed the following morning. Pregnant females, identified by the presence of a vaginal plug, were re-caged and offered 10 g/day of allocated diet throughout pregnancy. At 17.5 days gestation, dams were killed for collection of blood and organs.

### 2.2. Sample Collection

Animals were anesthetised using gaseous isoflurane, blood was removed by cardiac puncture and animals were killed by cervical dislocation. Blood was collected and stored in EDTA tubes. Whole blood 5-methyltetrahydrofolate (THF) and 5–10-methylTHF concentrations were measured by liquid chromatography-tandem mass spectrometry (LC-MS/MS) and data reported previously [25]. The uterus, containing all fetuses and placentas, was removed and placed immediately in ice cold PBS. The liver and placenta of each fetus were removed, weighed and snap frozen in liquid nitrogen and stored at −80 °C until required for RNA extraction.

### 2.3. RNA Extraction

To avoid any potential influence of sex on resultant data, only male tissues were analysed. Fetal sex was determined by polymerase chain reaction (PCR) of the sex determining region-Y (*SRY*) gene using DNA extracted from embryonic tail tissue [25,27]. RNA was extracted from whole fetal livers and placentas of males only using Tri-reagent (Sigma-Aldrich, Gillingham, Dorset, SP8 4XT, UK) and following the manufacturer’s instructions. Briefly, 50 mg tissue was homogenised in 500 μL Tri-reagent on ice. A further 500 μL Tri-reagent and 200 μL chloroform were added, then the sample was mixed by inversion and incubated on ice for 5 min. Samples were then centrifuged at 13,500 rpm for 15 min at 4 °C. The upper aqueous phase was removed, RNA was precipitated by incubation on ice with 500 μL isopropanol for 10 min, after which it was collected by centrifugation at 13,500 rpm for 10 min at 4 °C. The supernatant fluid was removed and the pellet was washed with 1 mL 75% ethanol for 10 min before centrifuging at 6000 rpm for 5 min at 4 °C. The supernatant fluid was removed, and the pellet was allowed to dry for 10 min before resuspending the RNA in 40 μL water. Contaminating DNA was removed using RQ1 DNase (Promega, Southampton, Hampshire, SO16 7NS, UK). RNA integrity was checked on an agarose gel and concentration and purity were measured using a Nanodrop spectrophotometer (Thermo Fisher, Waltham, MA, USA).

### 2.4. Gene Expression Arrays

RNA from each tissue was pooled for three male fetuses per litter (5 μg/fetus with a final concentration of 500 ng/μL) and hybridised to a single array for a total of 12 litters (*n* = 6 per dietary group) for each organ. Where a litter had more than three male fetuses, the three fetuses with weights closest to the mean litter weight were analysed.

Genome-wide transcript abundance was determined by ServiceXS (Plesmanlaan 1/D, 2333 BZ Leiden, The Netherlands) on the Affymetrix GeneChip platform with the NuGO mouse array (NuGO_Mm1a520177). This array comprises over 24,000 probe sets, covering the majority of established genes. Before the labelling process, the integrity of all RNA samples (RNA Integrity Number (RIN) > 8) was confirmed using the Agilent 2100 Bioanalyser (Agilent Technologies, Stockport, Cheshire, SK8 3GY, UK). Output data were supplied as Affymetrix CEL files and imported into R (version 2.15.3) using the Affy package [28]. Data from liver and placenta were pre-processed separately using gcRMA background correction and quantile normalisation [29] to correct for batch effects and other technical confounders. To maximize sensitivity and specificity, updated Entrez gene probe-set annotation was used from the BrainArray project [30] (version 14.1.0) resulting in 16,270 re-annotated probesets mapping to unique transcripts. Statistical analysis comparing the two diet groups was performed separately for each organ using the empirical Bayes approach of the Limma package [31] which performs a moderated *t*-test. On filtering genes for significant changes using False Discovery Rate (FDR) values calculated using the Benjamini and Hochberg method (FDR < 0.05), no genes remained statistically significant. Pathway analysis can in part replace FDR corrections by testing for the regulation of related genes, which suffers less from oversampling. Genes were, therefore, considered to show a differential expression relevant for further analysis in enrichment analysis in response to maternal folate depletion if there was a significant (*p* < 0.05) increase or reduction of at least 1.2 fold. DAVID [32] was used to carry out Gene Ontology enrichment analysis and to investigate KEGG pathways affected by maternal folate depletion. The threshold for significance for Gene Ontology enrichment analysis was set at *p* < 0.05 (corrected for multiple testing), and at *p* < 0.05 (uncorrected) for KEGG pathway enrichment analysis. Additional pathway analysis was carried out using PathVisio 3.2.0 and the curated pathway collection of WikiPathways (download date: 1 September 2015), applying the same parameters for significant fold-change as stated above, imposing a Z score of 1.9 for significance to filter for probable changed pathways. All raw and processed microarray data have been deposited in the ArrayExpress database (accession ID E-MTAB-3940).

### 2.5. Validation of Gene Expression Changes Using Real-Time PCR

To confirm the gene expression changes observed in the microarray analysis, real-time PCR was performed on each individual (i.e., not pooled) fetal RNA sample that was analysed by microarray hybridisation, focusing on 13 gene targets. Target genes were selected on the basis of being the most up or down regulated in the liver and placenta. Gene transcripts analysed in placental RNA were *Cyp21a1*, *Hbb-y*, *Slco1b2*, *Ptgs2*, *Vcan*, *Lrrn4*, and *Mettl7b* and in fetal liver RNA were *Asgr1*, *Hamp*, *Actc1*, *Ckm*, *Tnnc2* and *Smpx*.

RNA (1 μg) was reverse transcribed using Quantitect Reverse Transcription kit (Qiagen, Cat No. 205313) according to the manufacturer’s instructions. Briefly, 1 μg of RNA was incubated with 2 μL genomic DNA Wipeout buffer in a reaction volume of 14 μL at 42 °C for 2 min to remove any genomic DNA. The mixture was then placed immediately on ice. RNA was incubated with 1 μL Quantiscript reverse transcriptase, 1 μL RT primer mix and 4 μL Quantiscript RT buffer for 15 min at 42 °C, after which time it was incubated at 95 °C for 3 min. The cDNA samples generated were then diluted 1:9 with water for use in real time PCR.

Prior to sample analysis, expression values, linearity and efficiency of each assay were determined through cDNA standard curves completed for each transcript measured. Transcript levels of the genes of interest were measured on a Roche Lightcycler 480 (Roche Applied Science, Burgess Hill, West Sussex, RH15 9RY, UK) in a total reaction volume of 25 μL using 2.5 μL Quantitect sybr green transcript specific primers (Qiagen, see Supplementary Materials Table S1 for details of manufacturer’s individual catalogue numbers), 12.5 μL Quantitect SYBR green mix (Qiagen Cat. No. 204145), 1 μL diluted cDNA and 9 μL water and cycling parameters 95 °C for 5 min (1 cycle); 95 °C for 10 s followed by 60 °C for 30 s (40 cycles); followed by a final melt curve analysis and cooling to 40 °C. Using the delta CT method, transcript levels of the genes of interest were normalised to *GAPDH* transcript levels which were determined using the same procedure and parameters described.

### 2.6. In Silico Analysis of Gene Promoter Regions for Transcription Factor (TF) Binding Sites

Genomatix software (https://www.genomatix.de/v3.5 24 July 2015) was employed to obtain promoter sequences for genes of interest using the Gene2Promoter function. Promoter sequences were analysed for common transcription factor binding sites using the Common TFs function. Lists of TFs belonging to the transcription factor binding site families identified were downloaded from Genomatix and compared with fetal liver gene expression data.

### 2.7. Statistical Analysis

Statistical analysis of array data is described above. For all other datasets, data distributions were examined by the Kolmogorov-Smirnov test and all datasets were normally distributed. Analysis of variance (Statistical Package for the Social Sciences (SPSS) version 21, IBM, Armonk, New York 10504, NY, USA) was used to examine the effects of diet on placental weight, placental efficiency and gene expression analysis on data obtained from RT PCR analysis. *p* < 0.05 was considered statistically significant.

## 3. Results

### 3.1. Influence of Maternal Folate Intake during Pregnancy on Fetal Weight, Liver Weight, Placental Weight and Placental Efficiency

Data on the effects of maternal folate depletion on fetal weight and fetal liver weight have been presented previously [25]. In addition, exposure to the Low Folate diet reduced maternal whole blood 5’methyl THF concentration significantly (*p* < 0.001) with values of 666 (40.9) and 378 (50.0) nmol/L for Normal and Low Folate diets respectively [25]. Maternal folate depletion increased fetal weight but had no effect on weight of the fetal liver. There was no significant influence of maternal folate intake during pregnancy on placental weight (mean placental weights were 107.0 mg and 107.3 mg for normal (*n* = 56) and low (*n* = 37) folate groups respectively (*p* = 0.945)). Placental efficiency, calculated by dividing fetal weight by placental weight, was not influenced by maternal folate depletion (mean placental efficiencies were 7.9 and 9.1 for normal (*n* = 56) and low (*n* = 37) folate groups respectively (*p* = 0.200)).

### 3.2. Influence of Maternal Folate Intake during Pregnancy on Gene Expression in the Fetal Liver

In the fetal liver, 989 genes were differentially expressed in response to maternal folate depletion, comprising 555 up-regulated genes and 434 down-regulated genes (Supplementary Materials Table S2). All raw and processed microarray data have been deposited in the ArrayExpress database (accession ID E-MTAB-3940). Seventy three (7.4%) of these differentially expressed genes code for known transcription factor proteins (see Supplementary Materials Table S3 for list of genes). Although the majority (95.3%) of the gene expression changes were small (i.e., ranging from a fold change of 1.2–2), the expression of 47 genes was changed by greater than two-fold (Table 1).

Whilst gene ontology analysis found no statistically significant influence on biological processes, KEGG pathway analysis revealed that “Lysosome” and “Tight Junction” pathways were significantly affected in the fetal liver in response to maternal folate depletion (Table 2). Analysis of WikiPathways using PathVisio found 13 pathways in which a significant number of genes had altered expression in the fetal liver in response to maternal folate depletion (Table 3).

### 3.3. Influence of Maternal Folate Intake during Pregnancy on Gene Expression in the Placenta

In the placenta, 460 genes were differentially expressed (250 up-regulated and 255 down-regulated) in response to maternal folate depletion (Supplementary Materials Table S4). All raw and processed microarray data have been deposited in the ArrayExpress database (accession ID E-MTAB-3940). Twenty two (4.8%) of these differentially expressed genes code for known transcription factor proteins (see Supplementary Materials Table S5 for list of genes). Of all the genes expressed differentially, only three had a change in expression of greater than two-fold (Table 4), with the vast majority (99.4%) of changes being small (i.e., ranging from 1.2 to 2 fold change).

Whilst gene ontology analysis found no statistically significant effects on biological processes in placenta, KEGG pathway analysis revealed that “Amino sugar and nucleotide sugar metabolism”, “Valine, leucine and isoleucine degradation” and “Complement and coagulation cascades” pathways were significantly affected in the placenta in response to maternal folate depletion (Table 5). Interrogation of WikiPathways using PathVisio found 13 pathways which were altered significantly in response to maternal folate depletion (Table 6).

### 3.4. Comparison of Gene Expression Changes in the Placenta and Fetal Liver in Response to Maternal Folate Depletion

**Figure 1:**
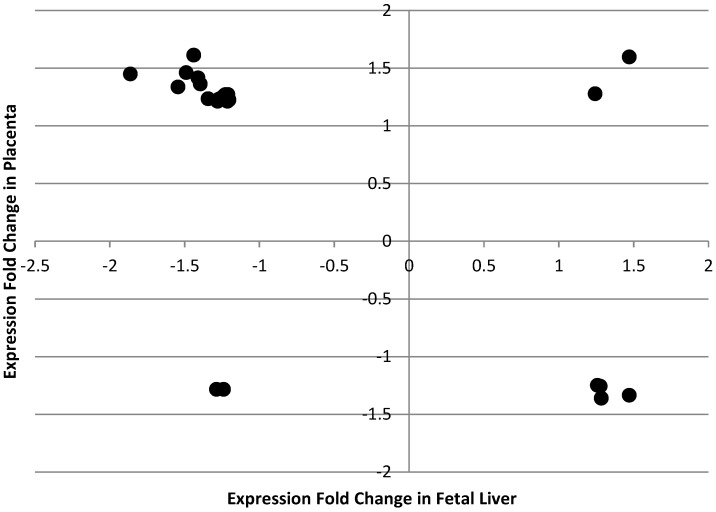
Scatterplot summarising the direction and level of fold change in expression of genes which were altered in both fetal liver and placenta by maternal folate depletion ^1,2,3^. ^1^ The Low and Normal folate diets contained 0.4 and 2.0 mg folic acid/kg diet respectively; ^2^ For each comparison, data were obtained from three male fetuses from each of six litters from dams fed either the Low or Normal folate diets; ^3^ A negative fold change indicates lower expression in offspring from dams fed the Low folate diet.

More genes in the fetal liver than in the placenta had altered expression in response to maternal folate intake during pregnancy (*n* = 989 and 460 respectively). Only 25 genes were common to the data-sets for fetal liver and placenta (see Table 7 for gene list). The expected overlap between two random subsets with elements of 989 and 460 respectively picked from a set of 16270 elements (the total number of measured genes) is 28, hence the observed overlap of 25 genes is likely due to chance. Of the genes found to respond to maternal folate in both placenta and fetal liver, only four displayed the same direction of change in both organs, with 21 showing the opposite direction of change (Figure 1), which suggests that finding expression changes in the same genes in the two organs may be due to chance.

No common KEGG pathways were significantly influenced by maternal folate intake in both the liver and placenta (Table 2 and Table 5). Use of PathVisio to interrogate WikiPathways revealed that “Alanine and aspartate metabolism” may be altered in both the fetal liver and the placenta of offspring of folate depleted mothers (Table 3 and Table 6). However, different genes in the pathway were differentially expressed in response to maternal folate depletion in the two tissues. In the fetal liver *Agxt* and *Pcx* were up-regulated in response to maternal folate depletion, whilst in the placenta *Abat* and *Asl* were down regulated.

### 3.5. Validation of Gene Expression Data from Array Analysis by RT-qPCR Analysis

For a subset of genes from both liver and placenta which showed altered expression in response to maternal folate depletion using microarray analysis, data were validated by RT-qPCR. For fetal liver, where the largest fold changes in response to maternal folate depletion were found in down regulated genes, we selected the four most down regulated genes i.e., *Actc1*, *Ckm*, *Tnn2* and *Smpx*. In addition, we analysed the two most up regulated genes i.e., *Hamp* and *Asgr1*. All genes showed the same direction of change using both methods (Table 8) but for 5/6 genes measured by RT-qPCR the change in expression was not statistically significant.

For placenta, fold changes in expression for up- and down-regulated genes in response to maternal folate depletion were similar and so the three genes with the largest fold changes in each direction were selected for validation analysis i.e., *Cyp21a1*, *Hbb-y* and *Slco1b2* for down-regulated genes and *Ptgs2*, *Vcan* and *Lrrn4* for up-regulated genes. In addition, a gene of particular interest, *Mettl7b*, was also selected for RT-PCR analysis. This gene encodes the protein methyltransferase-like 7B, and is likely to have methyltransferase activity (inferred through sequence similarity with other protein methyltransferases (http://www.uniprot.org/uniprot/Q6UX53)), and thus may have a role in epigenetic regulation of gene expression. All seven genes showed the same directional changes in expression in response to maternal folate depletion when assayed by RT-qPCR and by microarray but for 6/7 genes measured by RT-qPCR the change in expression was not statistically significant (Table 9).

### 3.6. Comparison of Gene Expression Changes in Fetal Liver in Response to Maternal Folate Depletion with Similar Published Transcriptomic Studies

Previous studies have reported gene expression changes at the transcriptomic level in the liver of rodents in response to diets low in folate [33,34,35]. Although several aspects of design differ between studies (summarised in Table 10), to identify a key set of genes in the liver that respond to changes in folate intake (or more broadly, changes in one carbon metabolism), we compared reported changes in gene expression between our data and three other studies [33,34,35]. Of the 989 genes which were differentially expressed in the fetal liver in response to maternal folate depletion in our study, 4, 89, 90 and 239 genes were also found to be differentially expressed in CBA mice, BALB/c mice, CBA/Ca mice and Wistar rats respectively [33,34,35] (full list of these genes for individual studies are in Supplementary Materials Table S6). Comparison of the differentially expressed genes from all studies revealed a set of 21 key genes whose expression was affected by altered folate/methyl donor intake in at least four of the five data sets (see Table 11). Importantly, none of the 25 genes which we found were expressed differentially in both fetal liver and placenta in response to maternal folate depletion are in this list of 21 genes.

### 3.7. In Silico Analysis of Promoter Regions of Key Folate/Methyl Donor Responsive Genes for Transcription Factor Binding Sites

For these 21 genes found to be affected robustly in liver by altered folate supply (Table 11), 76 unique promoter regions were found for 130 transcripts using the Gene2Promoter function in Genomatix (data not shown). Transcription factor binding sites for five families of transcription factors were present in 90% of the promoters investigated; ETS1 factors (ETSF), Kruppel like transcription factors (KLFS), Nuclear receptor subfamily 2 factors (NR2F), RXR heterodimer binding site (RXRF) and SOX/SRY sex/testis determining and related HMG box factors (SORY). Of these, the RXRF transcription factors had binding sites in all 76 analysed promoters. TFs belonging to the five identified families were compared with TFs found to be differentially expressed in the fetal liver and five TFs were found to overlap viz. *Elk3*, *Nr2c1*, *Nr2f1*, *Rxra*, *and Sox30.*

However the same analysis on randomly-selected sets of 21 genes that were affected by folate supply from each of the five studies plus two sets of 21 genes that did not respond to maternal folate supply revealed similar occurrence rates for these same transcription factor binding sites. Thus, this analysis identified no potential transcriptional regulatory mechanisms which may be responsible exclusively for coordinating the liver-specific changes in expression of these key genes in response to folate supply.

## 4. Discussion

Here we describe gene expression changes at the transcriptome level in the placenta and fetal liver in response to maternal folate depletion before mating and throughout pregnancy. Ames’ triage theory posits that if availability of a specific nutrient is inadequate, Darwinian processes ensure that essential functions, i.e., those required for short-term survival and/or reproduction, dependent on that nutrient that are protected at the expense of those functions that are less essential i.e., where reduced function does not have short term negative consequences. However, such prioritisation of some most critical (short-term) functions may impact adversely on other functions which may have long-term insidious effects that increase risk of diseases associated with ageing [23,24]. In response to inadequate maternal folate intake during pregnancy, we hypothesised that, due to different organ-specific functions, individual fetal organs would implement differential prioritisation hierarchies and thus display differential gene expression changes in response to folate depletion. We chose to investigate gene expression in the liver and placenta as examples of organs with very different roles in mammalian function across the lifecourse. In addition, the liver represents a major site for folate storage and metabolism, whilst the placenta (containing both maternal and fetal cells) is the route for delivery of nutrients (including folate) from the dam to the fetus during development. Our observations support our hypothesis that specific fetal organs exhibit specific transcriptional responses to maternal folate depletion. It is plausible that these organ-specific changes in gene expression are due to differential pathway prioritisation in each organ which reflect their contrasting functions.

The primary function of the placenta is the transfer of nutrients to the fetus, so it is essential that this function is protected to support fetal growth and development. In response to maternal folate depletion, we saw no changes in pathways associated with nutrient transport, suggesting that placental transfer of nutrients to the fetus was protected despite the reduced maternal folate intake. We observed no teratogenic effects of this reduced nutrient supply on the offspring [25]. It is important to note that we analysed whole placental samples containing both maternal and fetal cell populations and so we are unable to determine the cellular origin of the observed expression changes.

The liver is a highly metabolic organ responsible for a wide range of functions that are important at all life stages and include defence of the body against xenobiotics, assisting with digestion and with metabolism of absorbed nutrients, regulating blood lipids, synthesis of a wide range of secreted proteins and hormonal regulation. As such, during periods of undernutrition, it may not be possible for the liver to maintain all important functions, so that those functions essential for short-term survival and/or reproduction may be prioritised. The sequela is that some functions not critical for short-term survival but which are important for long term health may be de-prioritised leading to impaired function which may have significant long-term adverse effects for the organism. We observed that maternal folate depletion altered several processes and pathways, each of which could impact adversely on long-term liver function and organismal health. These included “adipogenesis genes”, “fatty acid biosynthesis” and “iron homeostasis” pathways. Whilst only one time point was investigated here (so that we are unable to comment on long-term gene expression changes or phenotypic outcomes), these observations are consistent with the fact that diets deficient in folate and choline can induce non-alcoholic fatty liver disease (NAFLD) in rodent models [36]. We observed up-regulation of genes involved in “fatty acid biosynthesis” (5/5 differentially expressed genes present on the pathway), and an abundance of up-regulated genes involved in the “adipogenesis genes” pathway (10/16 differentially expressed genes present on the pathway). Up-regulation of these genes may result in overall up-regulation of the pathways that potentially could result in altered hepatic fatty acid metabolism in offspring. Indeed, we observed that when the offspring of folate depleted dams were fed a high fat diet from weaning [37], they had significantly increased plasma triacylglycerol (TAG) concentrations whilst plasma TAG concentrations were unaffected in the offspring of folate replete dams [37]. Taken together, these data suggest that maternal folate depletion results in an altered response to high fat feeding which may be via programmed transcriptional changes in pathways associated with fat metabolism in the liver.

This analysis has focussed on the genes which were differentially expressed in the liver and in the placenta in response to maternal folate depletion. However, it is important to recognise that there are alternative explanations for genes which did not change in response to this nutritional insult. Whilst such unaltered genes may be components of pathways which are especially critical to that particular organ, it is also possible that these genes are simply unresponsive to folate supply. The present study design is unable to distinguish between these alternatives.

It is important to highlight that most of the gene expression changes observed in this study were relatively small and not statistically significant when applying the more stringent FDR value so that these data should be interpreted with caution. However, our observations are consistent with findings from other studies [33,34] and confirms the subtle, but pervasive, effects on gene expression of folate/methyl donor depletion. Furthermore, it is pertinent to point out that our gene expression studies were undertaken in male offspring only and it is possible, if theoretically unlikely, that effects could be different in females.

Whilst we show clearly that folate responsive gene expression changes are organ specific, the mechanisms behind the observed organ specific expression changes are less clear. A plausible mechanistic explanation could be based on inter-organ differences in expression of transcription factors (TFs) when exposed to inadequate folate intake. We tested this hypothesis by identifying all TFs which were differentially expressed in the liver and in the placenta in response to maternal folate depletion. In the fetal liver, we found that expression of 73 TFs differed in folate replete vs. folate depleted offspring, which accounted for 7.4% of the altered genes. In contrast, in the placenta only 22 TFs (4.8% of altered genes) displayed altered expression. Consistent with the idea that organ-specific changes in gene expression profiles are driven by organ-specific changes in TF expression, there was very little overlap between the TFs that were altered in liver and placenta—only one TF, *Med6*, was common between the organs.

It is plausible that changes in epigenetic processes are responsible for the organ-specific patterns in gene expression in response to folate depletion. Epigenetic marks, including DNA methylation, are modifiable by diet and other environmental factors and constitute an important and flexible system for regulating gene expression [38,39]. Folate is central to one-carbon metabolism and the formation of the universal methyl donor SAM, which is critical for the methylation of biological molecules including DNA. Indeed the influence of dietary folate intake on DNA methylation patterns has been reported widely [25,34,40,41,42] and may be responsible for the organ specific folate responsive gene expression changes observed here.

Although previous studies have reported gene specific expression changes in the rat placenta in response to increased levels of folate in the maternal diet [42,43], to the best of our knowledge this is the first report describing gene expression changes in response to low maternal folate (or methyl donor) intake in this organ, and the first study to investigate this at the transcriptomic level. However, previous studies have used transcriptomic approaches to investigate gene expression changes in the liver in response to low folate-containing diets [33,34,35,36,44,45] so we compared our data with outcomes from these studies. Whilst there were differences in gene expression profiles between studies, likely to be due to differences in rodent models, diet composition, duration and timing of the nutrient insult and the particular transcriptomic approaches employed (Table 10), we uncovered a key set of genes which were differentially expressed in the liver in response to low dietary folate/methyl donor intake across multiple studies (Table 11). Importantly, expression of none of these genes was altered in placenta by maternal folate depletion, indicating that these changes reflect a liver-specific response rather than being genes that are generally malleable in response to perturbation in folate supply. Moreover, this observation substantiates our suggestion that the transcriptome responses of fetal liver and placenta to folate restriction are distinct and physiologically-appropriate. Of the key set of 21 genes differentially expressed across multiple studies (Table 11), the use of DAVID [32] to carry out Gene Ontology enrichment analysis suggested that three genes are involved in wound healing i.e., *JUB*, *ENTPD2* and *SERPINE1* and, thus, altered transcription of genes associated with wound healing may help explain the observed association between low methyl donor intake and liver damage [36]. Furthermore, the discovery of these key folate responsive genes may provide candidate biomarkers for future studies of folate adequacy. Such studies should test the effects of different doses of folate without potential confounding from other dietary factors which may have been an issue in some of the studies included in Table 10.

To investigate potential mechanisms through which expression of these key genes in the liver are altered in response to methyl donor intake, the promoter regions were interrogated for the presence of common transcription factor binding sites. Whilst binding sites for five families of transcription factors were found to be highly represented in the promoters of these 21 genes, similar occurrence rates of these same transcription factor binding sites were also observed when seven randomly selected sets of genes were analysed. This suggests that this specific set of TFs is not responsible for co-ordinating transcriptional responses to maternal folate/methyl-donor supply in the liver. However, it is possible that there are regulatory regions outside the promoters of the genes investigated here which would merit investigation in future studies.

## 5. Conclusions

The data presented here suggest that maternal folate status during pregnancy influences gene expression in the fetus in a highly organ-specific manner. We hypothesise that this organ specificity is a rational response to limited nutrient supply which protects the most essential functions of each individual organ for short term survival (and reproduction), at the expense of less immediately essential processes which may lead to long-term adverse health sequelae. However, as we have noted above, there are viable alternative explanations for our observations. Our observations are consistent with the DOHaD hypothesis and suggest a plausible mechanism by which inadequate folate supply during early development leads to changes in gene expression to protect functions of the liver that are appropriate in this particular developmental and nutritional context, but that may contribute to the aetiology of cardiometabolic diseases if the postnatal environment is such that these changes become disadvantageous.

## Figures and Tables

**Table 1:** Genes with greater than two-fold expression change in fetal liver in response to maternal folate depletion ^1,2^.

Gene Symbol	Gene Name	Ensembl ID	Direction of Change	Fold Change	*p* Value	FDR
*Actc1*	actin, alpha, cardiac muscle 1	ENSMUSG00000068614	Down	20.5	0.001	0.379
*Ckm*	creatine kinase, muscle	ENSMUSG00000030399	Down	14.8	0.001	0.379
*Tnnc2*	troponin C2, fast	ENSMUSG00000017300	Down	14.0	0.015	0.458
*Smpx*	small muscle protein, X-linked	ENSMUSG00000041476	Down	13.4	0.001	0.379
*Tnni2*	troponin I, skeletal, fast 2	ENSMUSG00000031097	Down	9.1	0.016	0.460
*Actn2*	actinin alpha 2	ENSMUSG00000052374	Down	8.6	0.001	0.379
*Atp1b4*	ATPase, (Na^+^)/K^+^ transporting, beta 4 polypeptide	ENSMUSG00000016327	Down	7.9	0.009	0.443
*Tnnt3*	troponin T3, skeletal, fast	ENSMUSG00000061723	Down	7.6	0.016	0.461
*Eno3*	enolase 3, beta muscle	ENSMUSG00000060600	Down	7.5	0.003	0.379
*Tnni1*	troponin I, skeletal, slow 1	ENSMUSG00000026418	Down	7.2	0.008	0.435
*Myh8*	myosin, heavy polypeptide 8, skeletal muscle, perinatal	ENSMUSG00000055775	Down	6.9	0.024	0.487
*Casq2*	calsequestrin 2	ENSMUSG00000027861	Down	6.7	0.001	0.379
*Mybpc1*	myosin binding protein C, slow-type	ENSMUSG00000020061	Down	6.6	0.022	0.477
*Myh3*	myosin, heavy polypeptide 3, skeletal muscle, embryonic	ENSMUSG00000020908	Down	6.6	0.015	0.458
*Ldb3*	LIM domain binding 3	ENSMUSG00000021798	Down	6.3	0.002	0.379
*Mylpf*	myosin light chain, phosphorylatable, fast skeletal muscle	ENSMUSG00000030672	Down	6.1	0.021	0.477
*Sln*	Sarcolipin	ENSMUSG00000042045	Down	6.0	0.017	0.465
*Myl4*	myosin, light polypeptide 4	ENSMUSG00000061086	Down	5.4	0.005	0.407
*Ckmt2*	creatine kinase, mitochondrial 2	ENSMUSG00000021622	Down	4.9	0.007	0.435
*Myot*	Myotilin	ENSMUSG00000024471	Down	4.6	0.014	0.451
*Csrp3*	cysteine and glycine-rich protein 3	ENSMUSG00000030470	Down	4.3	0.017	0.465
*Srl*	Sarcalumenin	ENSMUSG00000022519	Down	4.1	0.005	0.407
*Pgam2*	phosphoglycerate mutase 2	ENSMUSG00000020475	Down	3.6	0.015	0.458
*Atp2a1*	ATPase, Ca++ transporting, cardiac muscle, fast twitch 1	ENSMUSG00000030730	Down	3.6	0.042	0.545
*Tnnt1*	troponin T1, skeletal, slow	ENSMUSG00000064179	Down	3.4	0.023	0.481
*Sh3bgr*	SH3-binding domain glutamic acid-rich protein	ENSMUSG00000040666	Down	3.1	0.002	0.379
*Itgb1bp2*	integrin beta 1 binding protein 2	ENSMUSG00000031312	Down	2.9	0.023	0.481
*Cdh11*	cadherin 11	ENSMUSG00000031673	Down	2.6	0.012	0.447
*Crh*	corticotropin releasing hormone	ENSMUSG00000049796	Down	2.5	0.036	0.529
*Fhl1*	four and a half LIM domains 1	ENSMUSG00000023092	Down	2.5	0.002	0.379
*Apobec2*	apolipoprotein B mRNA editing enzyme, catalytic polypeptide 2	ENSMUSG00000040694	Down	2.4	0.009	0.442
*Egfl6*	EGF-like-domain, multiple 6	ENSMUSG00000000402	Down	2.4	0.004	0.395
*Myom1*	myomesin 1	ENSMUSG00000024049	Down	2.4	0.029	0.498
*Mlf1*	myeloid leukemia factor 1	ENSMUSG00000048416	Down	2.3	0.020	0.476
*Cav2*	caveolin 2	ENSMUSG00000000058	Down	2.3	0.001	0.379
*Shh*	sonic hedgehog	ENSMUSG00000002633	Down	2.2	0.019	0.465
*Slit2*	slit homolog 2 (Drosophila)	ENSMUSG00000031558	Down	2.2	0.003	0.379
*Rps6ka6*	ribosomal protein S6 kinase polypeptide 6	ENSMUSG00000025665	Down	2.1	0.034	0.526
*Scn7a*	sodium channel, voltage-gated, type VII, alpha	ENSMUSG00000034810	Down	2.1	0.020	0.476
*Sparcl1*	SPARC-like 1	ENSMUSG00000029309	Down	2.1	0.012	0.447
*Nr1h4*	nuclear receptor subfamily 1, group H, member 4	ENSMUSG00000047638	Up	2.0	0.001	0.379
*Pemt*	phosphatidylethanolamine *N*-methyltransferase	ENSMUSG00000000301	Up	2.1	0.021	0.477
*Serpine1*	serine (or cysteine) peptidase inhibitor, clade E, member 1	ENSMUSG00000037411	Up	2.1	0.001	0.379
*LOC100503019*	hypothetical protein LOC100503019	NA	Up	2.5	0.018	0.465
*Cyp2e1*	cytochrome P450, family 2, subfamily e, polypeptide 1	ENSMUSG00000025479	Up	2.7	0.012	0.443
*Asgr1*	asialoglycoprotein receptor 1	ENSMUSG00000020884	Up	2.8	0.010	0.443
*Hamp*	hepcidin antimicrobial peptide	ENSMUSG00000050440	Up	3.3	0.001	0.379

^1^ The Low and Normal folate diets contained 0.4 and 2.0 mg folic acid/kg diet respectively; ^2^ For each comparison, data were obtained from three male fetuses from each of six litters from dams fed either the Low or Normal folate diets.

**Table 2:** KEGG pathways in the fetal liver altered by maternal folate depletion during pregnancy ^1^.

KEGG Pathway Term	Pathway Name	Total Genes on Pathway	Number of Altered Genes	Differentially Expressed Genes	*p* Value
mmu04142	Lysosome	119	12	*Ap3m1* (ENSMUSG00000021824), *Gm2a* (ENSMUSG00000000594)	0.031
				*Atp6v0a1* (ENSMUSG00000019302), *Naga* (ENSMUSG00000022453)	
				*Arsg* (ENSMUSG00000020604), *Gba* (ENSMUSG00000028048)	
				*Cd68* (ENSMUSG00000018774), *Ap3s2* (ENSMUSG00000063801)	
				*Gla* (ENSMUSG00000031266), *Dnase2b* (ENSMUSG00000028185)	
				*Galns* (ENSMUSG00000015027), *Gaa* (ENSMUSG00000025579)	
mmu04530	Tight junction	135	13	*Tjp1* (ENSMUSG00000030516), *B230120H23Rik* (ENSMUSG00000004085)	0.033
				*Myh3* (ENSMUSG00000020908), *Hcls1* (ENSMUSG00000022831)	
				*Gnai3* (ENSMUSG00000000001), *Myh8* (ENSMUSG00000055775)	
				*Nras* (ENSMUSG00000027852), *Actn2* (ENSMUSG00000052374)	
				*Ppp2r1a* (ENSMUSG00000007564), *Epb4.1l2* (ENSMUSG00000019978)	
				*Mylpf* (ENSMUSG00000030672), *Spnb2* (ENSMUSG00000020315),	
				*Akt2* (ENSMUSG00000004056)	

^1^ The Low and Normal folate diets contained 0.4 and 2.0 mg folic acid/kg diet respectively.

**Table 3:** WikiPathways in the fetal liver altered by maternal folate depletion during pregnancy ^1,2^ and identified using PathVisio.

Pathway	Number of Genes Altered on Pathway	Number of Genes Measured on Pathway	Total Number of Genes on Pathway	% Affected	Z Score	*p*-Value (Permuted)
Striated Muscle Contraction	16	32	46	50	9.35	<0.001
Adipogenesis genes	16	111	133	14	2.95	0.005
Fatty Acid Biosynthesis	5	21	26	24	2.93	0.011
Iron Homeostasis	3	10	16	30	2.77	0.013
TGF Beta Signaling Pathway	7	42	53	17	2.36	0.015
miR-1 in cardiac development	1	2	6	50	2.33	0.029
One carbon metabolism and related pathways	6	37	86	16	2.11	0.028
EPO Receptor Signaling	4	21	27	19	2.09	0.038
Spinal Cord Injury	11	85	110	13	2.04	0.035
Alanine and aspartate metabolism	2	8	43	25	1.94	0.038
Methylation	2	8	15	25	1.94	0.045
Osteoblast	2	8	14	25	1.94	0.040

^1^ The Low and Normal folate diets contained 0.4 and 2.0 mg folic acid/kg diet respectively; ^2^ For each comparison, data were obtained from three male fetuses from each of six litters from dams fed either the Low or Normal folate diets.

**Table 4:** Genes with greater than two-fold expression change in the placenta in response to maternal low folate intake ^1,2^.

Gene Symbol	Gene Name	Ensembl ID	Direction of Change	Fold Change	*p* Value	FDR
*Cyp21a1*	cytochrome P450, family 21, subfamily a, polypeptide 1	ENSMUSG00000024365	Down	2.5	0.002	0.999
*Hbb-y*	hemoglobin Y, beta-like embryonic chain	ENSMUSG00000052187	Down	2.2	0.035	0.999
*Slco1b2*	solute carrier organic anion transporter family, member 1b2	ENSMUSG00000030236	Down	2.1	0.011	0.999

^1^ The Low and Normal folate diets contained 0.4 and 2.0 mg folic acid/kg diet respectively; ^2^ For each comparison, data were obtained from three male fetuses from each of six litters from dams fed either the Low or Normal folate diets.

**Table 5:** KEGG pathways in the placenta altered by maternal folate depletion during pregnancy ^1, 2^.

KEGG Pathway Term	Pathway Name	Total Genes on Pathway	Number of Altered Genes	Differentially Expressed Genes	*p* Value
mmu00520	Amino sugar and nucleotide sugar metabolism	44	5	*Gnpda2* (ENSMUSG00000029209), *Pgm2* (ENSMUSG00000025791), *Uap1* (ENSMUSG00000026670), *Gmppb* (ENSMUSG00000070284), *Pgm3* (ENSMUSG00000056131)	0.017
mmu00280	Valine, leucine and isoleucine degradation	46	5	*Mccc2* (ENSMUSG00000021646), *Oxct2a* (ENSMUSG00000076436), *Hsd17b10* (ENSMUSG00000025260), *Bckdhb* (ENSMUSG00000032263), *Abat* (ENSMUSG00000057880)	0.019
mmu04610	Complement and coagulation cascades	75	6	*Cd55* (ENSMUSG00000026399), *Serpina1b* (ENSMUSG00000071178), *F11* (ENSMUSG00000031645), *F2* (ENSMUSG00000027249), *C9* (ENSMUSG00000022149), *C3* (ENSMUSG00000024164)	0.026

^1^ The Low and Normal folate diets contained 0.4 and 2.0 mg folic acid/kg diet respectively; ^2^ For each comparison, data were obtained from three male fetuses from each of six litters from dams fed either the Low or Normal folate diets.

**Table 6:** WikiPathways in the placenta altered by maternal folate depletion ^1,2^ during pregnancy and identified using PathVisio.

Pathway	Number of Genes Altered on Pathway	Number of Genes Measured on Pathway	Total Number of Genes on Pathway	% Affected	Z Score	*p*-Value (Permuted)
Complement Activation, Classical Pathway	3	13	19	23	3.77	0.002
Statin Pathway	3	16	29	19	3.26	>0.000
Alanine and aspartate metabolism	2	8	43	25	3.25	0.007
Glucocorticoid & Mineralocorticoid Metabolism	2	9	27	22	3	0.016
Selenium metabolism/Selenoproteins	3	18	49	17	2.98	0.015
Alzheimers Disease	6	54	91	11	2.98	0.006
Nuclear Receptors	4	31	38	13	2.79	0.015
Glucuronidation	2	10	33	20	2.78	0.012
Arachidonate Epoxygenase Epoxide Hydrolase	1	3	13	33	2.76	0.037
Polyol pathway	1	3	12	33	2.76	0.024
Complement and Coagulation Cascades	5	54	64	9	2.24	0.015
Acetylcholine Synthesis	1	5	18	20	1.97	0.050
Aflatoxin B1 metabolism	1	5	11	20	1.97	0.037

^1^ The Low and Normal folate diets contained 0.4 and 2.0 mg folic acid/kg diet respectively; ^2^ For each comparison, data were obtained from three male fetuses from each of six litters from dams fed either the Low or Normal folate diets.

**Table 7:** Genes differentially expressed in both fetal liver and placental organs in response to maternal folate depletion ^1,2,3^.

Gene Symbol	Ensembl ID	Expression fold Change in Fetal Liver	*p* Value for Fetal Liver	Expression Fold Change in Placenta	*p* Value for Placenta
*A830035A12RIK*	NA	1.5	0.025	−1.5	0.019
*Aadat*	ENSMUSG00000057228	−1.3	0.033	−1.3	0.031
*Akr7a5*	ENSMUSG00000028743	1.2	0.023	−1.3	0.003
*Apon*	ENSMUSG00000051716	1.4	0.019	−1.4	0.021
*Atp6v0a1*	ENSMUSG00000019302	1.2	0.028	−1.3	0.007
*Atrnl1*	ENSMUSG00000054843	−1.3	0.013	1.3	0.008
*Bcas3*	ENSMUSG00000059439	1.2	0.034	−1.2	0.035
*Col15a1*	ENSMUSG00000028339	−1.3	0.013	−1.2	0.029
*Cyld*	ENSMUSG00000036712	−1.3	0.030	1.3	0.024
*E130309D02RIK*	ENSMUSG00000039244	1.2	0.015	−1.2	0.027
*Fkbp8*	ENSMUSG00000019428	1.2	0.045	−1.3	0.020
*Inpp1*	ENSMUSG00000026102	1.2	0.019	−1.2	0.026
*Itgb4*	ENSMUSG00000020758	1.3	0.027	−1.2	0.039
*Med6*	ENSMUSG00000002679	−1.4	0.009	1.3	0.029
*Nubpl*	ENSMUSG00000035142	1.6	0.014	1.5	0.040
*Oxct2a*	ENSMUSG00000076436	−1.3	0.024	1.5	0.004
*Pgm3*	ENSMUSG00000056131	1.2	0.045	−1.3	0.028
*Prtg*	ENSMUSG00000036030	1.3	0.026	1.2	0.048
*Rbp2*	ENSMUSG00000032454	1.5	0.036	−1.9	0.001
*Sepx1*	ENSMUSG00000075705	1.2	0.040	−1.3	0.026
*Slc13a3*	ENSMUSG00000018459	1.6	0.003	−1.4	0.018
*Slc39a5*	ENSMUSG00000039878	1.4	0.049	−1.4	0.037
*Slc48a1*	ENSMUSG00000081534	1.3	0.012	−1.2	0.041
*Slc6a13*	ENSMUSG00000030108	1.3	0.021	−1.2	0.048
*Tomm40*	ENSMUSG00000002984	1.4	0.021	−1.5	0.001

^1^ The Low and Normal folate diets contained 0.4 and 2.0 mg folic acid/kg diet respectively; ^2^ For each comparison, data were obtained from three male fetuses from each of six litters from dams fed either the Low or Normal folate diets; ^3^ A negative fold change indicates lower expression in offspring from dams fed the Low folate diet.

**Table 8:** RT-qPCR expression analysis of selected genes which were differentially expressed in fetal liver in response to maternal folate depletion ^1,2^ when assayed by microarray.

Gene Name	Mean Expression Low Folate Group	SEM	Mean Expression Normal Folate Group	SEM	*p* Value	Direction of Change in Agreement with Array
*Asgr1*	0.35	0.07	0.12	0.07	0.050	Yes
*Hamp*	4.00	0.75	1.73	0.75	0.058	Yes
*Actc1*	3.63	3.04	8.29	3.04	0.305	Yes
*Ckm*	1.12	0.95	3.30	0.95	0.134	Yes
*Tnnc2*	0.51	0.66	2.39	0.66	0.073	Yes
*Smpx*	0.11	0.09	0.38	0.09	0.066	Yes

^1^ The Low and Normal folate diets contained 0.4 and 2.0 mg folic acid/kg diet respectively; ^2^ For each comparison, data were obtained from three male fetuses from each of six litters from dams fed either the Low or Normal folate diets. Expression values are in arbitrary units calculated using the delta CT method; *GAPDH* was used as the reference gene. Expression levels were quantified in individual fetuses (*n* = 18 per group) with mean expression levels calculated for individuals litters (*n* = 6 per group) for use in statistical analyses to match procedures used for array data.

**Table 9:** RT-qPCR expression analysis of selected genes which were differentially expressed in placenta in response to maternal folate depletion ^1,2^ when assayed by microarray. Expression values are in arbitrary units calculated using the delta cycle threshold (CT) method. *GAPDH* was used as the reference gene. Expression levels were quantified in individual fetuses (*n* = 18 per group) with mean expression levels calculated for individuals litters (*n* = 6 per group) for use in statistical analyses to match procedures used for array data.

Gene Name	Mean Expression Low Folate Group	SEM	Mean Expression Normal Folate Group	SEM	*p* Value	Direction of Change in Agreement with Array
*Cyp21a1*	0.55	0.13	0.88	0.13	0.118	Yes
*Hbb-y*	0.39	0.16	0.83	0.16	0.076	Yes
*Slco1b2*	0.29	0.08	0.49	0.08	0.106	Yes
*Ptgs2*	0.31	0.03	0.22	0.03	0.030	Yes
*Vcan*	0.66	0.09	0.46	0.09	0.144	Yes
*Lrrn4*	0.05	0.01	0.03	0.01	0.118	Yes
*Mettl7b*	3.07	0.61	4.83	0.61	0.068	Yes

^1^ The Low and Normal folate diets contained 0.4 and 2.0 mg folic acid/kg diet respectively; ^2^ For each comparison, data were obtained from three male fetuses from each of six litters from dams fed either the Low or Normal folate diet.

**Table 10:** Overview of experimental designs of studies investigating the influence of folate/methyl donor intake on genome wide gene expression in the liver.

Study	Model	Diets	Period of Exposure	Arrays
This study	Male C57Bl6J mice	2 mg folic acid/kg OR	4 weeks prior to mating to 17.5 days gestation	NuGO mouse array (NuGO_Mm1a520177)
		0.4 mg folic acid/kg		
Champier et al. [33]	Male CBA mice	8 mg folic acid + 10 g of succinylsulfathiazole/kg OR	8–12 weeks of age	CodeLinkUniset Mouse Whole Genome bioarrays
		0 mg folic acid + 10 g of succinylsulfathiazole/kg		
Glen et al. [35]	Male BALB/c & CBA/Ca mice	Low methionine (0.18%, w/w) & lacking choline & folic acid OR	4–12 weeks of age	NimbleGen 12 × 135 K Mouse Expression Array
		Low methionine (0.18%, w/w) & supplemented with choline & folic acid		
Chen et al. [34]	Wistar rats	Standard food OR	1 month before pregnancy to 21 days post-weaning	Agilent Arrays
		Diet without vitamin B_12_ & folate		

**Table 11:** Genes which were differentially expressed in response to low dietary folate/methyl donor intake in the liver across multiple studies ^1^.

	Gene Differentially Expressed				
Gene Symbol	McKay et al.	Champier et al. [33]	Glen et al. [35] (BALB/c Mice)	Glen et al. [35] (CBA/Ca Mice)	Chen et al. [34]
*ACOT2*	Y	N	Y	Y	Y
*COL6A2*	Y	N	Y	Y	Y
*CSTB*	Y	N	Y	Y	Y
*DCTD*	Y	N	Y	Y	Y
*ENTPD2*	Y	N	Y	Y	Y
*FETUB*	Y	N	Y	Y	Y
*FGF21*	N	Y	Y	Y	Y
*IL7R*	Y	N	Y	Y	Y
*JUB*	Y	N	Y	Y	Y
*LTBP2*	Y	N	Y	Y	Y
*OXCT1*	Y	N	Y	Y	Y
*RHBDF1*	Y	N	Y	Y	Y
*RUNX3*	Y	N	Y	Y	Y
*SERPINE1*	Y	N	Y	Y	Y
*SLC15A3*	Y	N	Y	Y	Y
*SLC30A10*	Y	N	Y	Y	Y
*SLC39A5*	Y	N	Y	Y	Y
*SLC41A3*	Y	N	Y	Y	Y
*SLPI*	Y	N	Y	Y	Y
*SORCS2*	Y	N	Y	Y	Y
*TMIE*	Y	N	Y	Y	Y

^1^ For details of diet compositions, please see Table 10. Y = Yes; N = No.

## Supplementary Materials

The following are available online at http://www.mdpi.com/2072-6643/8/10/661/s1, Table S1: Details of manufacturer’s product codes for primers used to determine gene transcript levels by RT-qPCR, Table S2: List of genes differentially expressed in the fetal liver in reponse to low maternal folate. Genes were considered to be significantly differentially expressed in response to low maternal folate if (a) the *p* value for t-test between dietary groups was < 0.05 and (b) if there was a fold change of ±1.2, Table S3: List of genes encoding transcription factors differentially expressed in the fetal liver in reponse to low maternal folate. Genes were considered to be significantly differentially expressed in response to low maternal folate if (a) the *p* value for t-test between dietary groups was < 0.05 and (b) if there was a fold change of ±1.2. B1, Table S4: List of genes differentially expressed in the placenta in reponse to low maternal folate. Genes were considered to be significantly differentially expressed in response to low maternal folate if (a) the p value for t-test between dietary groups was < 0.05 and (b) if there was a fold change of ±1.2.D1, Table S5: List of genes encoding transcription factors differentially expressed in the placenta in reponse to low maternal folate. Genes were considered to be significantly differentially expressed in response to low maternal folate if (a) the *p* value for t-test between dietary groups was < 0.05 and (b) if there was a fold change of ±1.2, Table S6: Lists of genes found in in common with other published data sets. Genes found to be differentially expressed in response to maternal folate depletion in the fetal liver were compared to other published gene lists where diets low in folate were reported to alter gene expression in the liver.
